# Involving hard-to-reach populations is pivotal for the tailoring and implementation of an epidemiological study in cross-border communities of French Guiana and Suriname

**DOI:** 10.3389/fpubh.2023.1162705

**Published:** 2023-05-31

**Authors:** Roxane Schaub, M. Sigrid Mac Donald Ottevanger, Soeradj Harkisoen, Béatrice Pesna, Celine Duijves, Marieke Heemskerk, Thomas Polime, Edouard Tuaillon, Stephen Vreden, Mathieu Nacher

**Affiliations:** ^1^CIC AG/Inserm 1424, Centre Hospitalier de Cayenne Andrée Rosemon, Cayenne, French Guiana; ^2^UMR Tropical Biome and Immuno-pathophysiology, Université de Guyane, Cayenne, French Guiana; ^3^Foundation for Scientific Research Suriname (SWOS), Paramaribo, Suriname; ^4^Department of Medical Microbiology, University of Amsterdam, Amsterdam UMC, Amsterdam, Netherlands; ^5^Department of Medical Microbiology, University Medical Center Utrecht, Utrecht, Netherlands; ^6^Centres Délocalisés de Prévention et de Soins, Centre Hospitalier de Cayenne Andrée Rosemon, Cayenne, French Guiana; ^7^GGD Hollands Noorden, Alkmaar, Netherlands; ^8^Social Solutions, Paramaribo, Suriname; ^9^Wooko Makandie Foundation, Culemborg, Netherlands; ^10^Pathogenèse et Contrôle des Infections Chroniques, INSERM U1058, Centre Hospitalier Universitaire de Montpellier, Montpellier, France

**Keywords:** hepatitis B virus, public health, cultural differences, Amazon, research

## Abstract

**Background:**

Hard-to-reach, vulnerable and cross-border populations are often disproportionately affected by communicable diseases. Epidemiological data on viral hepatitis in French Guiana and Suriname are available for urban areas, but not for remote communities. The Maroni River, which separates FG and Suriname, is home to Tribal and Indigenous communities. Reaching these populations is challenging due to logistical constraints, cultural and language barriers, and mistrust of outsiders.

**Objectives:**

We aimed to conduct an epidemiological study of viral hepatitis [Maroni Hepatites Virales (MaHeVi)] in this remote and complex area. Here, we describe the operational hurdles and solutions required to achieve this.

**Methods:**

We undertook a preliminary assessment of the area with local community leaders and health workers to gain approval of MaHeVi, acceptance of blood sampling, and suggestions for adapting the study to cultural and logistical constraints. Anthropological assessments were conducted through focus groups and interviews with key individuals to assess knowledge, beliefs and risk factors for VH.

**Results:**

MaHeVi was well received by the local communities. The approval of the community leaders was crucial for the implementation and acceptance of the study. The main adaptations were hiring community health mediators to overcome cultural and language differences, using blotting paper instead of venipuncture for logistical and acceptability reasons, and adapting communication materials.

**Conclusion:**

Careful preparation and tailoring of the communication materials and research protocol have enabled the successful implementation of the study. This process could be replicated in this area and transferred to other complex contexts combining borders, logistical hurdles and populations requiring cultural adaptations.

## Background

Vulnerable, migrant and indigenous populations are at an increased risk of chronic hepatitis B virus (HBV) (1, 2) and chronic hepatitis C virus (HCV) infection (3–5). These populations are key in epidemiological context and lowering-and ultimately eliminating – the burden of communicable diseases, and are often disproportionately affected by communicable diseases (6–8). Low-income and lack of health insurance enhance the vulnerability of these populations (9). Rural populations also suffer from health inequities worldwide, having a diminished access to healthcare compared to urban populations (10). Furthermore, vulnerable populations are prone to view researchers as outsiders, feel distrust, which can lead to recruitment barriers and challenges, especially in Indigenous communities (11). Therefore, disease prevalence rates in hard-to-reach or vulnerable populations are often calculated by proxy, using data from blood bank donors or healthier than average participants.

**Figure 1:**
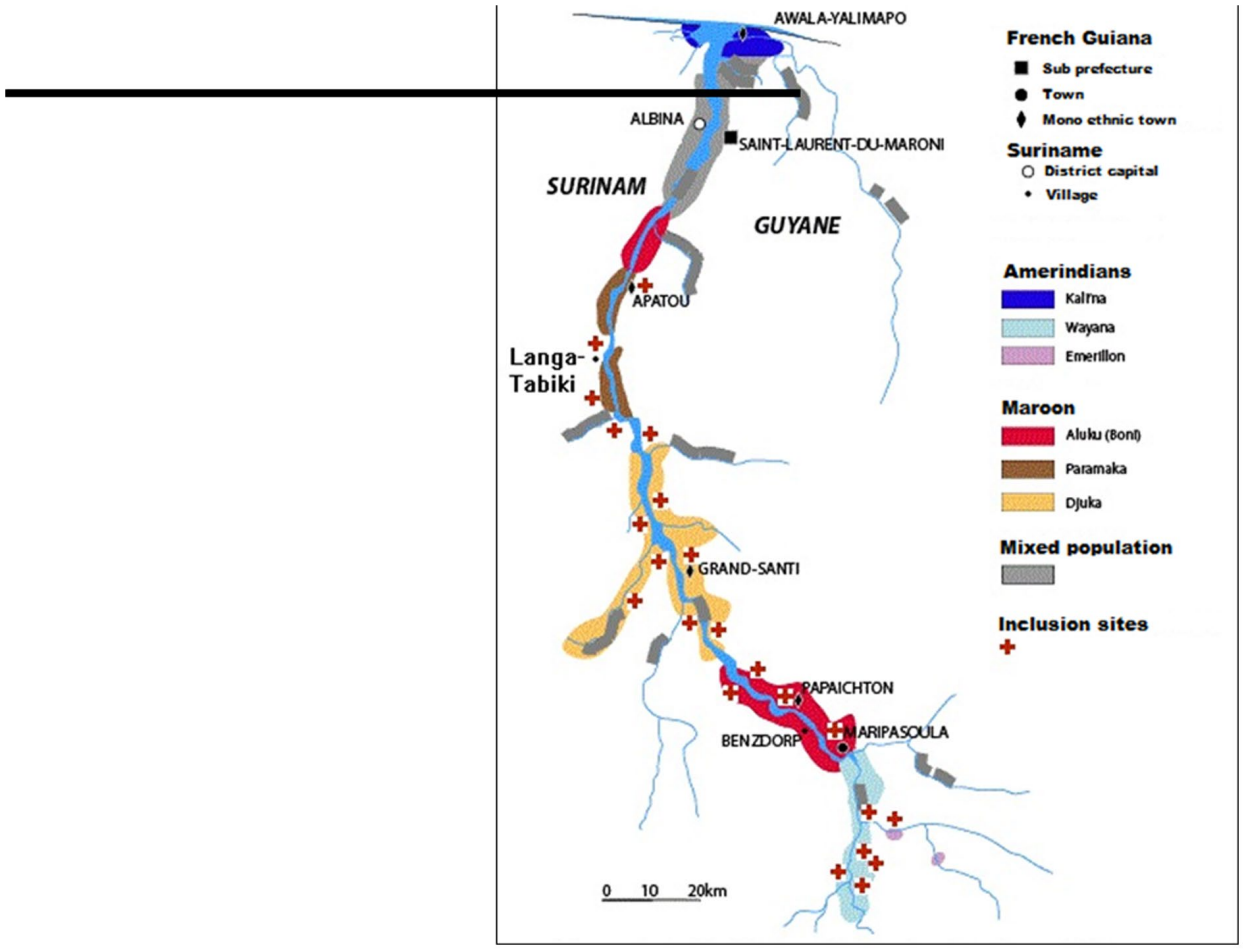
Map of the study region and the main population groups.

French Guiana (FG) and Suriname have a multi-ethnic and multicultural population reflecting their respective histories, including Tribal and Indigenous communities with a traditional lifestyle. Language, socio-economic level, education, and lifestyle vary widely depending on ethnicity and rural–urban location. The border between FG and Suriname – delineated by the Maroni river – is a remote, poorly accessible area, situated in the rainforest, home mainly to various Indigenous and Tribal communities, who generally have limited access to healthcare (12, 13) (Figure 1).

The Maroni river is mainly inhabited by Maroons (descendants from runaway enslaved individuals) and Amerindians (14), who live, respectively, on the northern and southern part of the river (Figure 1). The settlement of the different ethnic communities along the Maroni river follows historical events and natural boundaries formed by rapids that are difficult to cross, instead of administrative considerations (15). Except for Maripasoula and Saint-Laurent-du-Maroni which are multi-ethnic cities, the different communities live side by side in mainly mono-ethnic villages (14, 16, 17). Moreover, numerous gold-miner settlements deep in the forest contribute substantially to the migrant border population. These settlements concern thousands of mainly Brazilian gold-miners, but also traveling vendors, sex workers (SW) and others (18). Thus, despite the border, many people live alternatively in both countries and cross the border on a daily basis (19).

Each ethnic community has its own language, but most, apart from the Wayana, communicate together for daily life matters through *Mawinatongo*, the “language of the river.” Each community has their main leader, and each village their local leaders. People in these communities are often semi-nomadic, with frequent moves between villages (20). Men have a dominant role in both communities and most important public positions such as mayor, community leader, and village chief are held by men. On the French side, 5% of children aged 6–11 years and 8% of those aged 12–16 years do not attend school (21). In Suriname, 79.6% of Amerindian women and 45.3% of Maroon women have no or primary education (22). Traditional lifestyle predominates and most households have no electricity, running water or sanitation.

The Maroni basin is remote, with many transportation and communication problems. Apart from the main villages that concentrate the administrative, educational and health infrastructures, several hundred small settlements called *kampus* (about 100 on the Suriname side and 200 on the French side) – mostly remote and unregistered – are scattered along the river and its tributaries. These *kampus* are home to up to 50% of the inhabitants in parts of the Maroni basin. In FG, Maripasoula and Grand-Santi are accessible by plane and only Apatou is linked to the regional paved road network. In Suriname, most villages on the Maroni river are only accessible by air and/or by river (17). The journey may take up several hours depending on the river’s water level. In this area, FG and Suriname provide health care only through health clinics and medical mission posts in the main villages (23). Some of these clinics have doctors on duty, while others are staffed by nurses (Figure 1). Yet with health worker shortages, increasing costs, financial instability, political and social unrest, sustainable health care remains a challenge.

Data on viral hepatitis (VH) prevalence and VH health literacy in this border population are scarce. Available data in FG and Suriname are mainly confined to the urban areas: in Cayenne (FG), VH has strong ethnic clustering with HBV and HCV prevalence ranging between 0 and 11%, and 0 to 4.7% respectively, among various ethnic groups (24, 25). Similarly, in Paramaribo (Suriname), prevalence varies greatly, between 0.5 to 6.5% for HBV (26) and 0.6 to 4.1% for HCV (27).

### Objectives

We aimed to assess the burden of VH among populations living along this remote border between Suriname and FG. Their knowledge about hepatitis was assumed to be limited and therefore we needed to identify methods to inform, engage and finally test the hard-to-reach communities.

In this context, local teams in FG and Suriname tried to identify feasible and acceptable ways to assess knowledge, attitudes and beliefs (KAP-B) and study the epidemiology of VH in the river border-population between FG and Suriname—the aim of the MaHeVi study. The difficulties of implementing such a project in complex logistical and intercultural conditions are not unique to the context of the border between FG and Suriname. Therefore, here, we describe the operational hurdles and solutions required to implement our VH epidemiological study.

## Methods

### Study design

The MaHeVi study is a population-based, cross-sectional, non-interventional epidemiological study, whose main objective was to estimate the prevalence of HBV, HCV, Hepatitis D and HIV infection in the general adult population of the Maroni River basin upstream Apatou. The inclusion criteria were being 18 years or older, planning to stay on the Maroni within 2 months of inclusion, and signing informed consent to participate. The MaHeVi study (ClinicalTrials.gov, Identifier: NCT05002907) received ethics approval from the Ethics Committee of the Ministry of Health of Suriname (VG 023–16) and from the Institutional Review Board (IRB00003888) of the French Institute of Medical Research And Health.

In order to improve the feasibility and acceptability of the MaHeVi study, preliminary field missions were carried out to meet with local health actors (health centre staff and associations) and community leaders and authorities, to gather the support of community agents in communicating about VH and the study. In parallel, anthropologists assessed the acceptability of the study in the general population and collected information for the development of a relevant questionnaire, in particular on questions concerning body modifications and sexual and hygiene practices. They also assessed the criteria for involvement of the different communities in order to provide suggestions for improving acceptability and participation in the study.

### Preliminary field work for study adaptation

#### Awareness and communication with community leaders and community health care workers

The initial phase entailed an extensive communication campaign to engage the local community, starting with an audience with community leaders of the different ethnic communities and villages. These key persons were identified through local health centre managers and anthropologists. Topics covered were information on VH and possible risk-factors for transmission. We informed them about the purpose of the research project and requested their approval, acknowledgement and advice on how to proceed. The best ways to communicate about the study to potential participants and appropriate locations for inclusions were discussed with community leaders and local health care workers (HCWs) in each community. We also discussed the feasibility and acceptability of future participation concerning the process of inclusion, participation, reporting of results to participants and follow-up. We asked the community leaders for advice on how to carry out the project in accordance with local habits and customs.

Additionally, training on VH for HCWs was done prior to the study. The training was available for all HCWs from the health care centres, but also to the very rare private practitioners and pharmacists, and to employees and volunteers of local non-government organizations involved in healthcare. The training was conducted by specialists in VH from local hospitals, and covered general knowledge of VH (pathophysiology, epidemiology), management and treatment, and information on the MaHeVi study.

#### Anthropological studies

Preliminary qualitative anthropological studies were conducted in order to adapt the project to the local communities on different levels. First, to improve the understanding and acceptability of the project by exploring KAP-B on VH before implementation of the study. Second, we wanted to precisely identify and estimate the importance of various potential risk practices that are specific to these communities as well as the reality of known risk practices in these communities, in order to adapt the risk factors questionnaire in terms of accuracy, completeness, understanding and acceptability. Indeed, in these communities, there were reports of practices like homemade penile implants (28), dry sex (29), vaginal steam baths and multiple sexual partnerships (30, 31), which are suspected or confirmed risk factors for sexually transmitted infections. Polygamous marriage is traditional and widespread in the Maroon community, but not among Amerindians.

Two independent teams of anthropologists worked on each side of the river. The study sites were selected based on the ethnic groups residing in the villages/settlements. Triangulation, by involving multiple researchers and the combination of various data collection methods, was used to reduce bias and ensure the validity of the study.

In Suriname, two anthropologists and two assistants collected data in 3 villages and one Brazilian gold miners’ settlement (Antonio do Brinco), each with populations belonging to a different ethnic community (Figure 1). They performed 4 focus group discussions (FGD) per location, with a total of 120 participants, to obtain insights on common general behavioral patterns that people see around them: girls aged 15–20, women aged >20, boys aged 15–20 and men aged >20 years old, as well as 2 FGDs with SWs in Antonio do Brinco. The participants were selected by purposing sampling and the data collection was made with the help of brochures and picture cards and a problem tree exercise. Additionally, qualitative in-depth semi-structured interviews were conducted with 30 key persons and target individuals (health care workers, teachers, school leaders, and persons working in areas with a risk for transmission), to gain insight into personal knowledge and experiences with hepatitis and general insights of the community. The interviews explored knowledge, attitudes, behaviors around viral hepatitis, sexual and other practices that may lead to transmission.

In FG the anthropologist collected data in 12 sites, covering all ethnic communities (Figure 1). He used an ethnographic approach with 20 participatory observations, as well as 52 in-depth face-to-face semi-structured interviews with respondents aged ≥18, about their own experiences, and 27 interviews with informants with expertise in different aspects of the topic (teachers, HCWs, community spokespersons or chiefs, traditional practitioners, and SWs), all recruited through informants or acquaintances or during participatory observations. The anthropologists had an inductive approach to data analysis, meanings emerged from the data through exploration of different data sets, obtained from different sources (interviews, observations, documents) to generate a more comprehensive understanding in the analysis. Supplementary Tables S1–S3 show more details of the studied participants.

## Results

### Lessons from anthropological studies

KAP-B about VH varied widely among different populations, but overall knowledge was very low. Most people had never heard of hepatitis. Young people had more knowledge and understanding of sexual and health issues, having learned about them in biology classes, or from the school nurse. However, there were misconceptions about the modes of transmission of VH, including sharing of eating utensils and drinking glasses, and transmission through mosquito bites.

In the villages, anal and oral sex were somewhat taboo and uncommon, and men having sex with men occurred but was not talked about. In the gold mining settlements, neither oral nor anal sex was taboo. Commercial sex was not common in the villages, but available in nearby gold mining settlements. Focus group participants acknowledged the need for condoms but condoms were rarely used in steady relationships. In the Amerindian village, the negative impact of alcohol on condom use was explicit. In the gold mining settlements, condom use was reported to be consistent.

Sharing of toothbrushes, razors and other personal toiletries was not reported. Vaginal steam baths were very popular in Maroon communities and were used regularly. However, contrary to popular belief elsewhere, this practice in Maroon communities was not associated with sexual activity, but rather with an old tradition of achieving “cleanliness.” The term “dry sex” was considered offensive. Among Amerindian women, vaginal steam baths were only used after pregnancy.

For invasive skin procedures, earrings were always inserted with clean disposable needles. Other body piercings and tattoos were not always done professionally but participants often traveled to Paramaribo or FG to have them done. Piercings were particularly popular with young Amerindians. In the gold mining areas, cosmetic tattoos were popular and were often done in the country of origin. Aesthetic scarification is no longer practiced, but there is medicinal scarification called *koti*, where incisions on the forehead or back of the hand are coated with homemade medicines. Golden teeth, a status symbol, were mainly done in professional settings. Circumcision is not practiced.

Injection drug use was very rare in the area. Drug use was limited to smoking marijuana, crack and heroin, although “rape drugs” have recently been introduced in gold mining settlements.

Antenatal and postnatal care is available at health centres and includes VH and HIV screening for pregnant women and HBV vaccination at birth.

Following the preliminary anthropological studies, the anthropologists made recommendations on general organisation, communication and questionnaire content, which were implemented as far as possible for the MaHeVi study.

### Adaptation of MaHeVi communication, study design, and conduct

Following the preliminary steps, we adapted the MaHeVi study design and organisation on several ways, as summarized in Table 1.

**Table 1:** Recruitment barriers and challenges for the MaHeVi project, and resulting study adaptation.

Recruitment barriers and challenges	Study adaptation
Traditional social structure	Consultation of community leaders and local health workers prior to study
Potential distrust in community outsiders	Extended stay in each inclusion location to allow time for potential participants to become familiar with the project and the field team and working hand in hand with local health structures
Cultural differences between researchers and participants and between participants from different ethnic communities, language barrier	Hiring health mediators from the community, ideally not living in the same village as the one surveyed because of sensitive questions
Mostly oral communication and low literacy rate	Adaptation of recruitment material through audio communication on local radio stations and display communication promoting illustration
Acceptability of blood sampling and sample transportation	Choice of dried blood spot instead of tube blood sampling
Individual transportation difficulties for participants, due to transportation costs and remoteness	Travel of field teams instead of participants
Transborder situation involving administrative constraints and highly mobile potential participants	Operation based on 1 field team in each country, moving in a coordinated way in the territories of each community and using the same communication and identification

**Figure 2:**
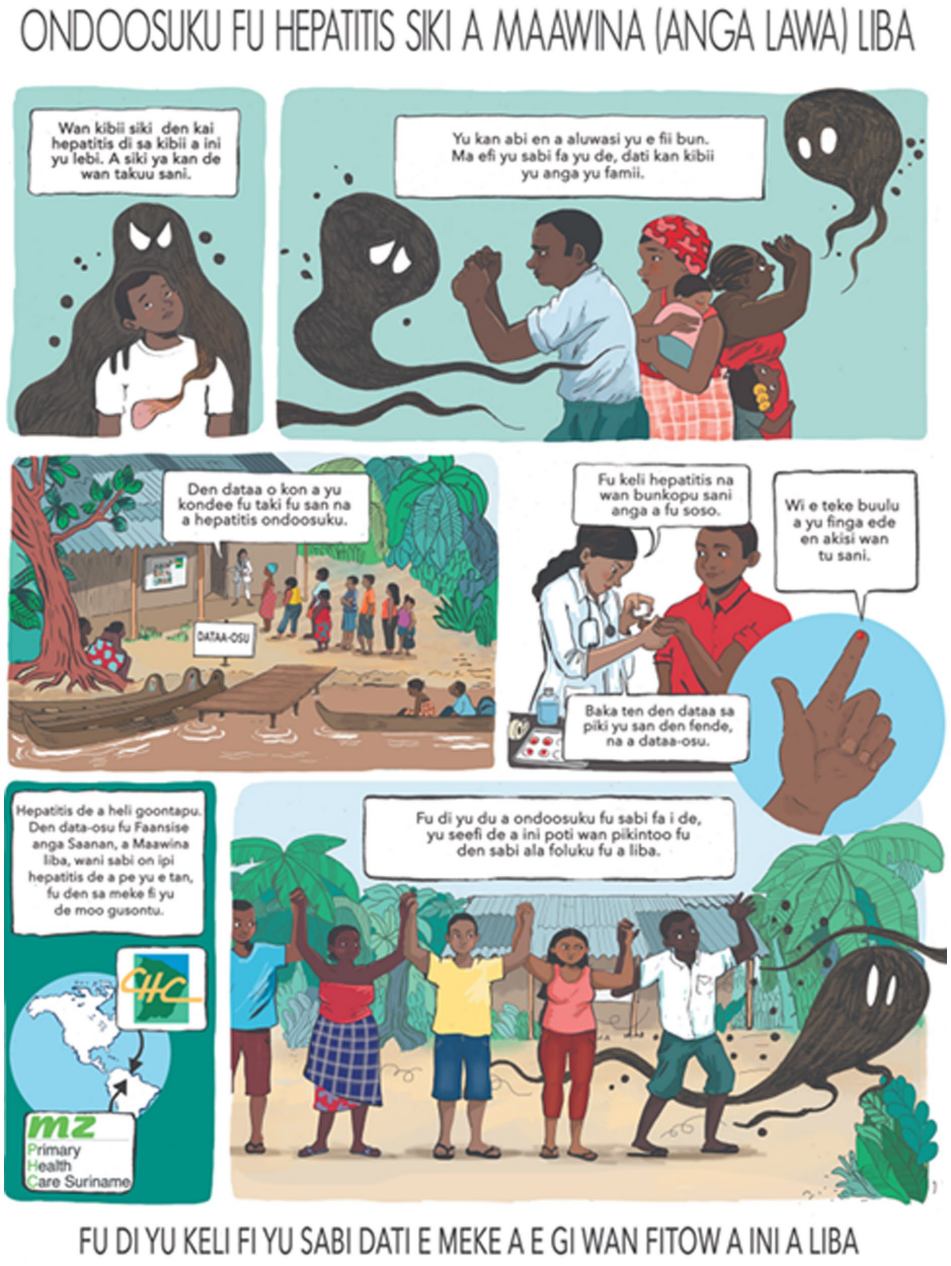
Posture used to introduce the MaHeVi project (Ndyuka version).

Discussions with local key persons, HCWs and non-government organizations, helped us to identify and anticipate communication difficulties linked to poor knowledge and awareness on VH, and low literacy. They suggested favoring oral communication with public meetings, live interviews and radio infomercials broadcasted on local stations, and poster campaigns including drawings rather than writing and allowing the population to identify with the characters. The posters were developed using the well-known logos of local health structures, and involved a not-for-profit organisation with experience of developing visual information materials with local communities. A poster was developed to introduce the MaHeVI project (Figure 2). These posters and flyers were distributed in key locations, e.g., health centres, shops and other community gathering places.

A short repeat communication campaign, including another visit to the local authorities and airing of radio-infomercials, kicked off the inclusions of participants in the MaHeVi study. Local radio stations announced the dates of arrival of MaHeVi teams on both sides of the border shortly before inclusions.

In addition to gaining the consent of community leaders, we improved the acceptability of the study by making a visible commitment to our cross-border partnership and to working with local health centres that people knew and trusted. The study therefore took place primarily in or near the health centres and usual places of regular health outreach, following local health workers on their regular outreach in collaboration with community volunteers, before moving further into the field where possible.

Trained health mediators had to be fluent in the language of the specific communities. In addition, as a result of preliminary discussions, mediators were required to have no relationship or connection with the village where inclusions were taking place, if possible, to ensure maximum participation and honest answers to questions. Participants often preferred non-locals to administer the questionnaires and report the results, as they were seen as less likely to divulge confidential information in the village.

We considered using fingerpricks and filter paper, i.e., DBS, rather than conventional blood sampling, due to limited or no access to centrifugation facilities and sufficient refrigerated storage space, coupled with uncertain logistics of repatriating samples. In addition, the cold chain could not be guaranteed because the electricity supply was intermittent, with only the larger settlements having access to a generator. In addition, people are used to capillary blood samples for rapid HIV and malaria testing.

Conventional geographical cluster sampling, which would have been more accurate in obtaining a more representative sample of the target population, was not feasible due to poor accessibility, especially in the hundreds of unregistered *kampus*. The necessary elements for a relevant random selection of participants were not available, either individually or by residential area. In addition, visiting a *kampu* directly could be perceived as intrusive. Not giving everyone the opportunity to participate could also be misinterpreted. We therefore decided to focus on communication, particularly radio broadcasts, and hypothesized that almost the entire target population would be informed about the study in an area where radio is popular and word of mouth is very effective. In order to compensate for the selection bias, we decided to adjust the sample according to the number of people included per community, since we did not refuse participants even if the target number of participants per community was reached, and opted for a post-stratification weighting method based on the population structure.

## Conclusion

### Lessons from the field

In the setting of the Maroni River, contrary to the widespread belief that epidemiological research would face insurmountable difficulties, MaHeVi was well accepted by the local community leaders and the people of the various communities on both sides of the border.

Following an initial field communication mission, the local authorities acknowledged the seriousness of the disease and agreed to the study and our presence in the area. In addition, the Granman of the Ndyuka Maroon community requested that the area of inclusion be extended to include the Tapanahony River, a tributary further inland in Suriname, in order to provide screening and treatment options for the entire community. The approval of the local community authorities was crucial for the acceptance and implementation of the study.

Focus group and key-person discussions within the various local populations were important for a tailored development of communication materials on VH, adaptation of the study questionnaire, as well as acceptance of the sample collection. The choice of DBS as the sampling method was mainly motivated by its indication in populational studies and its use in challenging settings (32). Furthermore, preference for DBS instead of venepuncture was confirmed in our study by several key persons, HCWs and community chiefs, but also by random respondents. In general, it was clear that this blood sampling method would increase acceptability to participate in the MaHeVi project.

The involvement of the local community in the adaptation of the study protocol, especially community leaders, as well as in the development and finalization of the posters and radio announcements, was essential for the communities’ commitment to the project, as has been showed elsewhere (33).

### Limitations

There are several limitations to this preliminary study. First, not all *kampus* are registered, possibly creating a selection bias in the people interviewed. However, we believe that working simultaneously on both sides of the border, involving all known ethnic groups in FGDs and interviewing local HCWs, community chiefs and authorities, provides an accurate reflection of the populations’ KAP-B.

Second, it is necessary to continue our efforts to go further in involving local communities in health interventions to ultimately improve health outcomes (34). In particular, for the epidemiological studies needed to establish appropriate and evaluable health studies and interventions, we need to improve the level of community participation and progress toward interactive participation (33). This requires the promotion of intercultural dialog between Western and Traditional communities, through study co-design and evaluation and the establishment of a sustainable climate of trust and partnership (35).

In conclusion, following the preliminary studies and subsequent adaptations, the inclusions in the MaHeVi study took place from 2018 to 2019. Enrollment of participants on both sides of the Maroni was completed as planned. The results of the seroprevalence and risk factors study will be published and communicated to the communities concerned.

## Data availability statement

The original contributions presented in the study are included in the article/Supplementary material, further inquiries can be directed to the corresponding author.

## Author contributions

All authors listed have made a substantial, direct, and intellectual contribution to the work and approved it for publication.

## Funding

The MAHEVI project was funded by the Programme de Coopération Interreg Amazonie FEDER/2016/N°189 and by the Agence Nationale de Recherche sur le VIH/SIDA et les Hépatites virales (ANRS 95025).

## Conflict of interest

The authors declare that the research was conducted in the absence of any commercial or financial relationships that could be construed as a potential conflict of interest.

## Publisher’s note

All claims expressed in this article are solely those of the authors and do not necessarily represent those of their affiliated organizations, or those of the publisher, the editors and the reviewers. Any product that may be evaluated in this article, or claim that may be made by its manufacturer, is not guaranteed or endorsed by the publisher.
