# Supplementary material for: Involving hard-to-reach populations is pivotal for the tailoring and implementation of an epidemiological study in cross-border communities of French Guiana and Suriname
Source: Front Public Health. 2023 May 31;11:1162705. doi: 10.3389/fpubh.2023.1162705 (PMC10266529; doi:10.3389/fpubh.2023.1162705)
Supplement: Supplementary file 1 [file Data_Sheet_1.docx]

Table S1. Overview of focus group discussion participants and key individuals among the different inclusion sites on the Suriname side of the Maroni

| Target group | Langatabiki | Stoelmanseiland/Gakaba | Kawemhakan |
| --- | --- | --- | --- |
| FGD females 15-20 | 5 | 8 | 4 |
| FGD males 15-20 | 4 | 11 | 3 |
| FGD females >20 | 8 | 10 (Gakaba) | 10 |
| FGD males >20 | 4 | 9 (Gakaba) | 7 |
| Target individuals interviewed | Shop owner (1) | Microscopist (2)  Traditional healer (1)  Military (2)  Shop owner Stoelmanseiland (1)  Shop owner Gakaba (2)  Taxi driver to gold mining area Gakaba (1) |  |

Abbreviation: FGD: focus discussion groups.

( ) the number of individuals interviewed are shown in parentheses.

Table S2. Focus Group participant description

| Location | Focus Group Discussion participants | Number of participants | Interviews |
| --- | --- | --- | --- |
| Antonio do Brinco | Dominican females SW, ages 25-35 | 5 | nail salons (2)  hair dressers/barbers (2)  Other area inhabitants (2) |
| Antonio do Brinco | Brazilian males, all ages (<20 to >50) | 9 |  |
| Antonio do Brinco | Dominican females SW, ages 20-35 | 4 |  |
| Peruano | Brazilian mixed gender group; 7 males, 3 Females, all ages | 10 |  |
| Peruano | Brazilian females SW, ages 20-35 | 4 |  |
| Peruano | Brazilian females SW and 1 male, ages 25-40 | 5 |  |

Abbreviations: FGD: focus discussion groups ; SW: sex workers

( ) the number of individuals interviewed are shown in parantheses.

Table S3: knowledge and perception of various risk factors among the different inclusion sites on the Suriname side of the Maroni

|  | Area | | | |
| --- | --- | --- | --- | --- |
| Risk factor | Langatabiki | Stoelmanseiland/Gakaba | Kawemhakan | Antonio do Brinco/Peruano |
| Anal/oral sex | Both somewhat a taboo. Not common in the village according to input.  Not familiar with MSM | Both somewhat a taboo. Not common in the village according to input.  Not familiar with MSM | Both somewhat a taboo. Not common in the village according to input.  MSM not talked about but occurs. | No taboo. Oral sex is ‘just part of it’ in a relation and during working hours. Anal sex is done by some of SW. |
| Paid sex | Not common in village. Cabarets in surroundings. | Not common in village. Cabarets in surroundings. | Not common in village. Cabarets in surroundings. | SW with various nationalities working in cabarets. |
| Condom use | Aware of necessity. Not used in relation. Focus on prevention from pregnancy. Women often ashamed to collect one. | Aware of necessity. Not used in relation. Focus on prevention from pregnancy. Women often ashamed to collect one. | Aware of necessity. Not used in relation. Focus on prevention from pregnancy. Women often ashamed to collect one. Excessive alcohol use perceived to have a negative impact on use of condoms. | Aware of necessity. SW use condoms consistently. Men and women have no problems collecting. |
| Boegroes (penile implants) | Popular among men of all ages. | Popular among men of all ages. | Not common | Not common |
| Earrings | No sharing of needles | No sharing of needles | No sharing of needles | No sharing of needles |
| Piercings | Parlours in Paramaribo and French Guiana. Not all professional. | Parlours in Paramaribo and French Guiana. Not all professional. | Parlours in Paramaribo and French Guiana. Not all professional. Popular among youngsters visiting school in French Guiana. | Piercings were obtained in home country or in Paramaribo. |
| Tattoos | In Paramaribo or French Guiana. Not all professional. | Some in village. Most often in Paramaribo or French Guiana. Not all professional. | Parlours in Paramaribo and French Guiana. Not all professional. Popular among youngsters visiting school in French Guiana. | Number of SW with tattooed eyebrows. Tattoos were obtained in home country or Paramaribo. |
| Scarification | Does not occur | Does not occur | Does not occur | Does not occur |
| Drug use | No intravenous drugs use.  Only smoking drugs. | No intravenous drugs use.  Only smoking drugs. | No intravenous drugs use.  Only smoking drugs. | No intravenous drugs use.  Only smoking drugs. New phenomenon is ‘rape drugs’. |
| Hairdressing | Hairdressers and individuals who do haircuts aware of hygiene and single use of razorblades. | Hairdressers and individuals who do haircuts aware of hygiene and single use of razorblades. | Hairdressers and individuals who do haircuts aware of hygiene and single use of razorblades. | Hairdressers and individuals who do haircuts aware of hygiene and single use of razorblades. |
| Manicure/pedicure | Does not occur. | Does not occur. | Does not occur. | High standards of hygiene and very aware of risks. |
| Sharing toothbrushes, razors and other personal toiletries. | Does not occur. | Does not occur. Only youngsters from boarding school share nail clippers occasionally. | Does not occur. | Does not occur. |
| Golden teeth | Not in the village. Only in professional locations. | Not in the village. Only in professional locations. | Not in the village. Only in professional locations. | Not in the settlements. Only in professional locations. |
| Mother to child transmission | Prenatal and postnatal care available in the village although women visit health clinic in French Guiana. | Prenatal and postnatal care available in the village although most women visit health clinic in French Guiana. | Prenatal and postnatal care available in the village although most women visit health clinic in French Guiana. | No prenatal and postnatal care available in the settlements. Most women visit health clinic in French Guiana. |
| Circumcision | Does not occur. | Does not occur. | Does not occur. | Does not occur. |
| Vaginal herbal steam baths | Popular. Intensity and regularity of the baths varies. | Popular. Intensity and regularity of the baths varies. | Only after pregnancy. | Use vaginal washes sold in the Brazilian pharmacies. |

Abbreviation: SW: sex workers
